# Transcriptome and Metabolome Analyses of the Salt Stress Response Mechanism in *Lonicera caerulea*

**DOI:** 10.3390/biology14060641

**Published:** 2025-05-31

**Authors:** Dandan Zang, Yadong Duan, Hengtian Zhao, Ning Wang, Yiming Zhang, Yanmin Wang, Huizi Liu

**Affiliations:** 1 Northeast Institute of Geography and Agroecology, Chinese Academy of Sciences, 138 Haping Road, Harbin 150081, China; zangdandan@iga.ac.cn (D.Z.); duanyadong@iga.ac.cn (Y.D.); zhaohengtian@163.com (H.Z.); wangning@iga.ac.cn (N.W.); 2 Huma Cold Temperature Plant Germplasm Resources Protection Field Scientific Observation and Research Station of Heilongjiang Province, Northeast Institute of Geography and Agroecology, Chinese Academy of Sciences, Da Hinggan Ling 165100, China; 3 Agriculture & Forestry Technology Cillegal, Weifang Vocational College, Weifang 261108, China; nefuzym@126.com; 4 Key Laboratory of Fast-Growing Tree Cultivating of Heilongjiang Province, Forestry Research Institute of Heilongjiang Province, Harbin 150081, China; 5 Zhejiang Institute of Subtropical Crops, Zhejiang Academy of Agricultural Sciences, Wenzhou 325005, China

**Keywords:** *Lonicera caerulea*, salt stress, physiological changes, RNA-seq, LC-MS

## Abstract

*Lonicera caerulea* is a wild fruit species with high edible and medicinal value. However, the molecular regulation and metabolic mechanisms of *L. caerulea* under salt stress are still unclear. Salt stress causes damage to the cell membrane of *L. caerulea* and induces changes in malondialdehyde content, relative electrolyte leakage, leaves’ stomatal opening, and the water loss rate. It also increases the activity of antioxidant enzymes and the content of soluble sugars. A comprehensive transcriptomic and metabolomic analysis of *L. caerulea* exposed to salt stress at four different (treatment) time intervals yielded a total of 99,574 unigenes and 1375 metabolites. Among these, 4081, 4042, and 4403 differentially expressed genes (DEGs) were identified in 12 transcriptomes, while 776, 832, and 793 differentially accumulated metabolites (DAMs) were detected in 12 metabolomes. The DEGs play important roles in several signaling pathways, including MAPK signaling, fatty acid metabolism, starch and sucrose metabolism, phenylpropanoid biosynthesis, and plant hormone signal transduction. KEGG pathway enrichment analysis revealed that these DEGs and DAMs are associated with flavonoid and lipid biosynthesis pathways. The combined transcriptomic and metabolomic analyses suggest that flavonoid and fatty acid compounds may be involved in regulating plant responses to salt stress. These findings will lay the foundation for the selection of *L. caerulea* germplasm resources and the expansion of its cultivation area. These research findings will lay the foundation for the cultivation of salt-tolerant new varieties of *L. caerulea* and their planting in saline–alkali soils.

## Simple Summary

This study explores how salt stress affects *Lonicera caerulea*. Salt stress damages cell membranes and alters various physiological traits, including antioxidant enzyme activity and soluble sugar content. Transcriptomic and metabolomic analyses identified thousands of differentially expressed genes and metabolites involved in key pathways like MAPK signaling, fatty acid metabolism, and flavonoid biosynthesis. These findings suggest that flavonoids and fatty acids play crucial roles in salt stress response. The results lay the groundwork for breeding salt-tolerant *L. caerulea* varieties suitable for saline–alkali soils.

## 1. Introduction

Soil salinization severely restricts the available arable land area and is also one of the major factors limiting high crop yields [1]. Approximately 10% of the global land area is affected by soil salinization [2]. In China, the problem accounts for almost 25% of the entire farmed land in the country, and the issue of soil salinity is growing increasingly serious as a result of irrational irrigation [3,4,5]. Soil salinization leads to an increase in the content of soluble salts in the soil, an elevation in soil osmotic pressure, and plasmolysis in plant root cells, which weakens the ability of plant roots to absorb water and nutrients, thereby affecting the normal growth and development of plants [6]. Soil salinization not only affects agricultural production but is also detrimental to the stability of ecosystems and biodiversity [7].

During their growth and development, plants experience various environmental changes, including biotic and abiotic stresses [8]. The series of physiological and ecological changes that plants undergo in response to abiotic stresses have become a focus of attention [9,10]. When plants are subjected to salt stress, osmosis and ion disorders will occur in plant cells, which will destroy the cytoplasmic membrane, lead to metabolic disorders, and affect the normal growth and development of plants [11]. To decrease the negative impacts of salt stress, plants have come up with diverse adaptation strategies to withstand salt stress [12,13].

Plants respond to salt stress by regulating their metabolic reactions, particularly through the synthesis of secondary metabolites. Secondary metabolites play important roles in antioxidant defense, cell protection, signal transduction, and resistance to pathogen invasion [14]. Plants maintain intracellular pH and ionic balance by accumulating organic acids [15]. Concurrently, levels of plant hormones like abscisic acid (ABA), salicylic acid (SA), and jasmonic acid (JA) fluctuate, playing roles in plant defense responses and signal transduction [16]. Furthermore, plants increase the production of antioxidants such as flavonoids and phenolic compounds to mitigate excessive reactive oxygen species (ROS) and shield cells from oxidative harm [17]. ROS, acting as secondary messengers in cellular metabolism, are crucial for plant growth; however, excessive ROS levels can induce undesirable programmed cell death [18,19].

RNA-Seq can be used to study the dynamic changes in gene expression and monitor the differential expression of genes under different temporal and spatial conditions. Such technology does not strictly require a control genome [20]; it has become convenient to study non-model species [21,22,23]. The analysis of RNA-Seq in rice roots under salt stress demonstrated that some DEGs were implicated in multiple signal transduction pathways under salt stress [24]. RNA-Seq analysis was performed on the top leaves of the young oil palm (*Elaeis guinensis* Jacq.) under salt stress, and seven significant DEGs were found. Homologous genes of the above seven genes were found in other species, and it was found that these seven genes indeed perform positive or negative regulation of salt tolerance, which is consistent with the RNA-Seq anticipated results [25]. We believe that the salt stress to which the plants are subjected has a significant impact on their physiology and gene expression.

Metabolomics provides direct information on metabolic changes in plants under salt stress. Through non-targeted metabolomics analysis, it can be found that the content of antioxidant metabolites such as arbutin increases exponentially in salt-tolerant plants, while it significantly decreases in sensitive varieties [26]. The changes in these metabolites reflect the physiological state and adaptation mechanisms of plants under salt stress, such as maintaining intracellular water balance by accumulating osmoregulatory substances or clearing excess ROS by enhancing the activity of the antioxidant system.

Combining transcriptomics and metabolomics research can provide a more comprehensive understanding of the molecular basis of plant salt tolerance. By analyzing DEGs and metabolites, the metabolic regulatory network of plants under salt stress can be revealed, and how these metabolic changes affect plant growth, development, and salt tolerance. This multi-omics joint analysis method is helpful in identifying key genes and metabolites related to salt tolerance, providing a theoretical basis for cultivating salt-tolerant crop varieties and potential solutions to the problem of salinization in agricultural production.

*L. caerulea* is a perennial shrub of the Lonicera family [27]. It is classified as a “homology of medicine and food” plant due to its high concentration in biologically active chemicals [28]. It also has high economic value and ornamental value, while it is antioxidant and cold-resistant [29,30]. The growth of *L. caerulea* is sensitive to environmental changes [31]. It grows strongest in slightly acidic soils, alkaline soil has a certain effect on the growth of *L. caerulea*, and altitude also restricts the growth and development of *L. caerulea* [32]. Saline soil is the main reserve soil resource in China, and about 80% of the saline soil has yet to be exploited [33,34]. Therefore, for the honeysuckle industry, how to cope with and adapt to the impact of soil salinization has become an urgent problem to be solved.

There have been few studies on the salt tolerance of *L. caerulea*, and there is a lack of research on the signaling network in reaction to salt stress [35,36,37]. In order to study the salt stress tolerance of *L. caerulea*, we determined the electrolyte leakage, starch, soluble sugar, flavonoids, sucrose, proline, and malondialdehyde contents of *L. caerulea* under 150 mM NaCl stress for 0, 12, 24, and 48 h, respectively. In addition, we determined activities of three antioxidant enzymes (POD, CAT, and APX) and leaves stomatal aperture of *L. caerulea* at four different (treatment) time intervals. By analyzing the physiological, transcriptomics, and metabolomics of *L. caerulea* under salt stress, salt-tolerant genes can be identified, and the physiological changes that these particular genes might influence were verified, leading to the creation of new salt-tolerant varieties, expanding the distribution range of *L. caerulea*, and improving the utilization efficiency of saline soil. We hope that our findings will shed more light on the molecular mechanism underlaying salt stress resistance and physiological changes in salt-resistant honeysuckle.

## 2. Materials and Methods

### 2.1. Plant Materials and Treatments

We used *L. caerulea* cutting seedlings to propagate the plants in the culture media containing sand and turf peat (1:2, *v*/*v*) in the greenhouse, maintaining a constant temperature of 24 °C, 70–75% relative humidity (RH), and light/dark pattern of 14 h/10 h. In the field of stress research on blue honeysuckle, the combination of 12 h, 24 h, and 48 h is commonly used as sampling time points. These time points can observe significant changes in plant physiology and gene expression while also balancing the feasibility of the experiment and the reliability of the results [38,39,40]. Studies on salt stress in honeysuckle plants have shown that 150 mM NaCl is a commonly used concentration [41,42]. Moreover, 150 mM NaCl can induce a salt stress response without causing excessive damage to the plant as high salt stress would. The two-month-old, well-watered seedlings were then treated with NaCl solution (150 mM) for 0, 12, 24, and 48 h [41,43,44,45]. The seedlings that received fresh water (150 mM NaCl for 0 h) were used as control. After the treatments, we collected the whole plants, separately (control, 12, 24, and 48 h), merged them into one sample (with 3 replicates of each), placed them in liquid nitrogen, and kept them at −80 °C.

### 2.2. RNA-Seq Analysis

Total RNA was extracted using the CTAB method and treated with DNase (Toyobo, Osaka, Japan). Biomaker (Beijing, China) was responsible for enriching mRNAs, sorting fragments, adding adapters, selecting sizes, and conducting PCR. We used HiSeq 2000 PE150 (Illumina, Inc., San Diego, CA, USA) for sequencing each library.

We removed low-quality results and adapter readings from raw sequences, including reads whose N percent was >5% or those that contained >15% nucleotides and had Q-values ≤ 19 (Q-value indicates Phred quality score). Thereafter, we adopted clean reads for transcriptome read-mapping and de novo assembly. We used Trinity software (version 20140717) for the de novo assembly of RNA-seq data. We then spliced unigenes into 12 libraries and utilized the Bowtie2 approach (version 2.2.3) to obtain non-redundant unigenes for subsequent analyses.

Functional annotations of unigenes include Open Reading Frame (ORF), GO, KEGG pathways, and the similarity of protein sequences. We identified ORF sequences of unigenes using TransDecoder (version 20140717) and conducted a sequence similarity search in protein databases (Nr, Swiss-Prot). Then, we predicted the protein functions as per its analogy with the closest proteins in the above-mentioned databases. The Blast2GO 6.0 software was utilized for GO functional annotation analysis [46]. The GO annotation of each unigene was obtained; then, WEGO 2.0 software was applied for each unigene to gain insights into its involvement with biological functions [47]. For unknown genes, we used the Cluster of Orthologous Group (COG) method. The prospective molecular pathways were investigated using the KEGG database.

To build DEG profiles, we analyzed read numbers in every coding region of all the 12 libraries, which were then converted into readings per kilobase exon model per million mapped reads (RPKM) [48]. For multiple tests and analyses, we adopted a false discovery rate (FDR) to determine the threshold *p*-value. Additionally, |log2Ratio| ≥ 1 and FDR ≤ 0.0001 were deemed thresholds for adjusting significant differences at gene levels among both the control and treatment groups. A Venn diagram was used to compare the expression of genes among the three treated groups of plants. DEGs were clustered together, and their fold changes (FCs) were noted. To ascertain the molecular functions of the expressed genes in treated plants, the DEG profiles of each were classified based on GO. Furthermore, the database of UniProt, Pfam, and KEGG was used for analyzing any significant functions of expressed genes.

### 2.3. Real-Time RT-PCR Analysis

Five randomly selected genes were subjected to qRT-PCR to validate the RNA-seq data. We carried out the qRT-PCR experiments and analyzed the data using the methods of Zang [35] and Livak [49]. Supplementary Table S1 lists the primers used in the PCR.

### 2.4. Physiological Analysis

All plants were used for a physiological analysis to determine gene functions involved in salt resistance. We measured the MDA level as described by Sheoran [50], while an electrolyte leakage experiment was conducted as described by Jambunathan [51]. Measurements of the activities of POD and CAT were conducted according to the description by Rao [52]. Measurements of the activities of SOD were conducted according to the description of Zhang [53]. The water loss rate was measured as the method described by Zhang [54]. The stomatal aperture of leaves was determined following a protocol [55]. Stomatal length and width were directly measured using the microscope’s built-in measurement tools, and the stomatal length-to-width ratio was calculated. The content of sucrose was determined using a Plant Sucrose Content Assay Kit (Solarbio, Beijing, China, BC2465). The content of starch was determined using a Starch Content Assay Kit (Solarbio, Beijing, China, BC0700). The content of flavonoids was determined using a Plant Flavonoids Content Assay Kit. (Solarbio, Beijing, China, BC1330). A measurement of the activities of APX was conducted according to the description of Hard [56]. The soluble sugar content was determined following a protocol [57]. Proline content was measured using the method described by Liu [58]. Three independent biological replications were performed.

### 2.5. Metabolome Analysis

Metabolomic analyses were performed on *L. caerulea* after salt stress at different time points. The instrument system for the metabolite data acquisition of the samples mainly consists of liquid chromatography (LC) (SHIMADZU Nexera X2, Tokyo, Japan) and tandem mass spectrometry (MS/MS) (Applied Biosystems QTRAP, Foster City, CA, USA). For each sample, 100 mg was taken for metabolite extraction, and 4 µL was used for quality control. The mass spectrometry peaks of metabolites were subjected to peak area analysis and correction. Characteristic ions were screened, and the peak area integration data were analyzed. Differential metabolites and related genes in the pathways were also analyzed. Principal component analysis (PCA) can be conducted as described by Wang et al. [59]. Orthogonal partial least squares discriminant analysis (OPLS-DA) can be performed following the procedure outlined by Thévenot et al. [60]. The analytical approach for Ultra-Performance Liquid Chromatography-Mass Spectrometry (UPLC-MS) (Milford, MA, USA) can be based on the methodology established by Want et al. [61].

### 2.6. Statistical Analysis

Data were tested with the variance (ANOVA) program, and statistical analysis was performed using the Statistical Package for the Social Sciences (version: SPSS 18.0). A one-way ANOVA was used for the analysis. Statistical significance was defined as a *p*-value ≤ 0.05.

## 3. Results

### 3.1. Physiological Changes in L. caerulea Exposed to Salt Stress at Four Different (Treatment) Time Intervals

**Figure 1:**
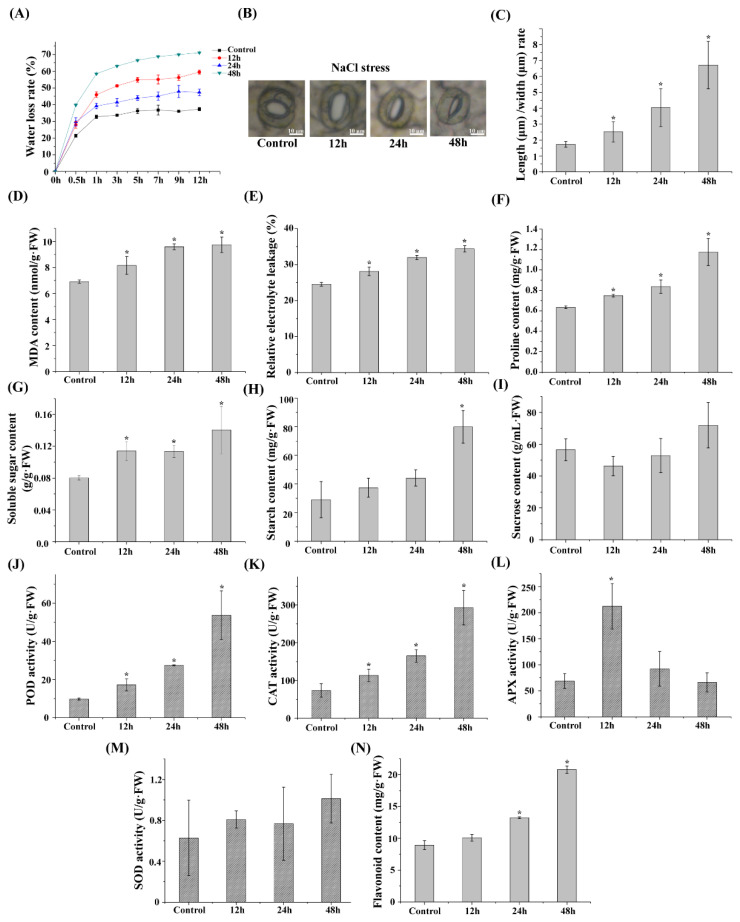
The impact of salt stress treatment at different times on plant physiological and ecology. (**A**) Measurement of water loss rate. (**B**,**C**) Determination of leaves stomatal aperture; (**D**) MDA content analysis; (**E**) analysis of relative electrolyte leakage; (**F**) measurement of proline content; (**G**) measurement of soluble sugar content; (**H**) measurement of starch content; (**I**) measurement of sucrose content; (**J**–**M**) analysis of antioxidant enzyme activity, involving POD activity (**J**), CAT activity (**K**), APX activity (**L**), and SOD activity (**M**); (**N**) determination the content of flavonoid. The data are the averages of three independent experiments. Error bars indicate the SD. * The significant difference (*t*-test, *p* < 0.05) compared with the control.

*L. caerulea* seedlings were treated with 150 mM NaCl for 12, 24, and 48 h (*n* = 3 replicates each), with untreated seedlings serving as the control. The whole plants of *L. caerulea* were then taken for transcriptome and metabolome analysis. At the same time, we examined the physiological changes of *L. caerulea* under salt stress. The water loss rate of *L. caerulea* leaves increased significantly with the extension of salt stress (Figure 1A). To clarify whether the change in water loss rate was related to the change in the stomatal length/width ratio, we investigated the changes in stomatal length and width under salt stress (Figure 1B). The study revealed that the stomatal length/width ratio increased with the extension of salt stress duration (Figure 1C). MDA levels and relative electrolyte leakage were significantly increased in *L. caerulea* at 12 h, 24 h, and 48 h compared to the control (Figure 1D,E). Proline, soluble sugar, and starch contents were significantly increased under salt stress, while sucrose content did not change significantly (Figure 1F–I). Under salt stress, POD and CAT enzyme activities increased with the prolongation of stress time, SOD activity changed insignificantly, and APX activity reached a peak at 12 h and then gradually decreased with prolonged stress duration (Figure 1J–M). In addition, the determination of flavonoid content under salt stress revealed that the flavonoid content in *L. caerulea* increased with the prolongation of salt stress time (Figure 1N).

### 3.2. Identification of DEGs

**Figure 2:**
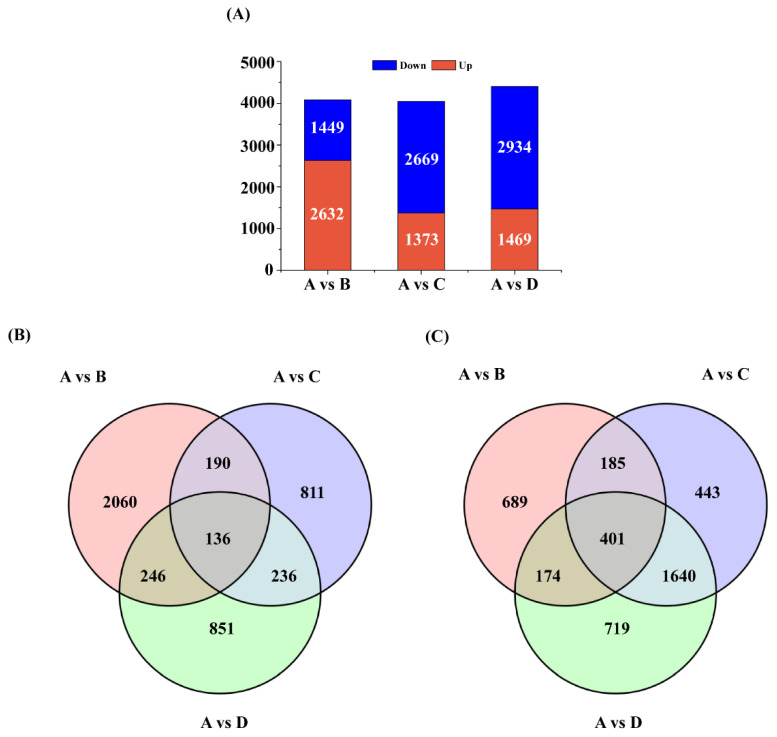
Overview of DEGs of *L. caerulea* under salt stress. (**A**) Total number of up-/down-regulated expressed genes at different treatment times. A vs. B represents the control vs. 12 h, A vs. C represents the control vs. 24 h, and A vs. D represents the control vs. 48 h. (**B**) A Venn diagram depicting the distribution of up-regulated and down-regulated DEG counts across the three gene sets. A vs. B represents the control vs. 12 h, A vs. C represents the control vs. 24 h, and A vs. D represents the control vs. 48 h. (**C**) A Venn diagram depicting the distribution of down-regulated DEG counts across the three gene sets. A vs. B represents the control vs. 12 h, A vs. C represents the control vs. 24 h, and A vs. D represents the control vs. 48 h.

DEGs in *L. caerulea* exposed to salt stress at four different time intervals (control, 12 h, 24 h, and 48 h) based on RNA-seq were identified. The results showed that there were 4081 DEGs after the control and 12 h of salt exposure. Among them, 2632 DEGs were up-regulated (UR), while 1449 DEGs were down-regulated (DR). Additionally, 4042 DEGs were obtained from plants treated for control and 24 h. Among them, 1373 DEGs were UR, and 2669 DEGs were DR. There were 4403 DEGs after control and 48 h salt exposure. Among them, 1469 DEGs were UR, and 2934 DEGs were DR (Figure 2A). Moreover, 326 and 586 genes were similarly up- and down-regulated both in the control and 12 h and 24 h treatments; 382 and 575 genes were up- and down-regulated in the same manner under 12 and 48 h salt stress, and 372 and 2041 DEGs were similarly up- and down-regulated when the authors compared the data after 24 h and 48 h salt treatment (Figure 2B,C). The above genes may be related to plant growth and development and can resist salt stress by regulating metabolic pathways. Only 136/401 unigenes were found to be similarly up/down-regulated among the control group and salt stress-treated groups (Figure 2B,C). These genes are the key genes for salt stress. The differentially regulated genes were subjected to hierarchical cluster analysis.

To verify the accuracy of Solexa sequencing, we conducted qRT-PCR on 7 DEGs previously identified by Solexa sequencing with respective primers. These 7 genes were selected randomly. The correlation coefficient between qRT-PCR and transcriptome data reached R^2^ = 0.83, indicating that the transcriptome sequencing results have high accuracy and reproducibility (Figure S1).

### 3.3. DEGs Are Highly Enriched in the Phenylpropanoid Biosynthesis Pathway

**Figure 3:**
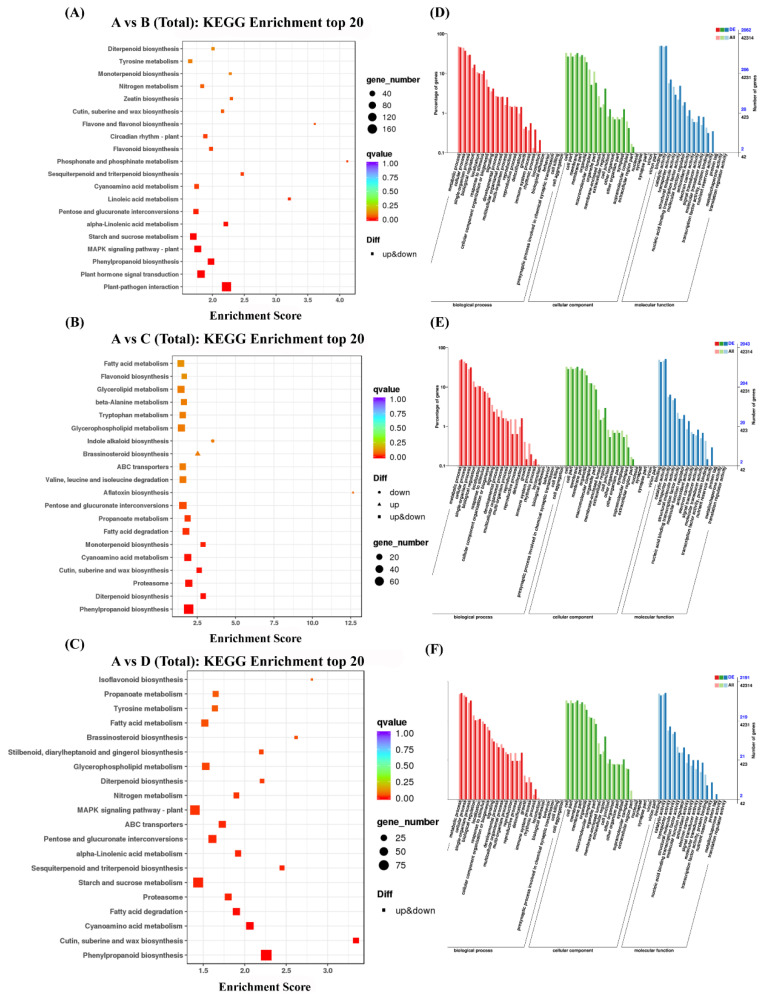
The top 20 KEGG pathway groups and GO classification of DEGs that were significantly enhanced during salt stress. (**A**–**C**) The bigger the enrichment factor, the greater the importance of DEGs in the pathway. The color of the circle symbolizes the q-value. The larger the q-value, the less credible the importance of the containing of DEGs in the pathway; The size of the circle represents the number of genes concentrated in the pathway; the smaller the circle, the fewer genes. A vs. B represents the control vs. 12 h, A vs. C represents the control vs. 24 h, and A vs. D represents the control vs. 48 h. (**D**–**F**) The Go classification of DEGs under salt stress.

DEGs were analyzed using KEGG techniques to clarify the major metabolic pathways of *L. caerulea* during salt stress. Figure 3 displays 20 KEGG pathways (*p* < 0.05). Compared with the control, DEGs were considerably increased in plant–pathogen interaction, plant hormone signal transduction, phenylpropanoid biosynthesis, the MAPK signaling pathway, and starch and sucrose metabolism after 12 h of salt stress treatment (Figure 3A). Compared with the control, at 24 h of salt stress treatment, DEGs were significantly enriched in phenylpropanoid biosynthesis (Figure 3B). Compared with the control, salt-stress-treatment 48 h DEGs were significantly enriched in phenylpropanoid biosynthesis. We found some similar pathways under different treatment times (Figure 3C).

DEG profiles obtained from salt-treated plants (for 0 h, 12 h, 24 h, and 48 h) were subjected to GO analysis to predict the functions of the expressed genes (Figure 3D–F). Most genes were enriched in biological process (BP) terms, while cell component (CC) and molecular function (MF) terms were ranked second and third, respectively. As for the CC terms, membrane and cell parts were the leading terms. As for BP terms, metabolic and cellular processes were the most significant ones. Concerning MF terms, catalytic activity and binding were the most prominent ones. Consequently, the above GO terms show significant molecular changes in the leaves of *L. caerulea* in response to salt stress conditions.

**Figure 4:**
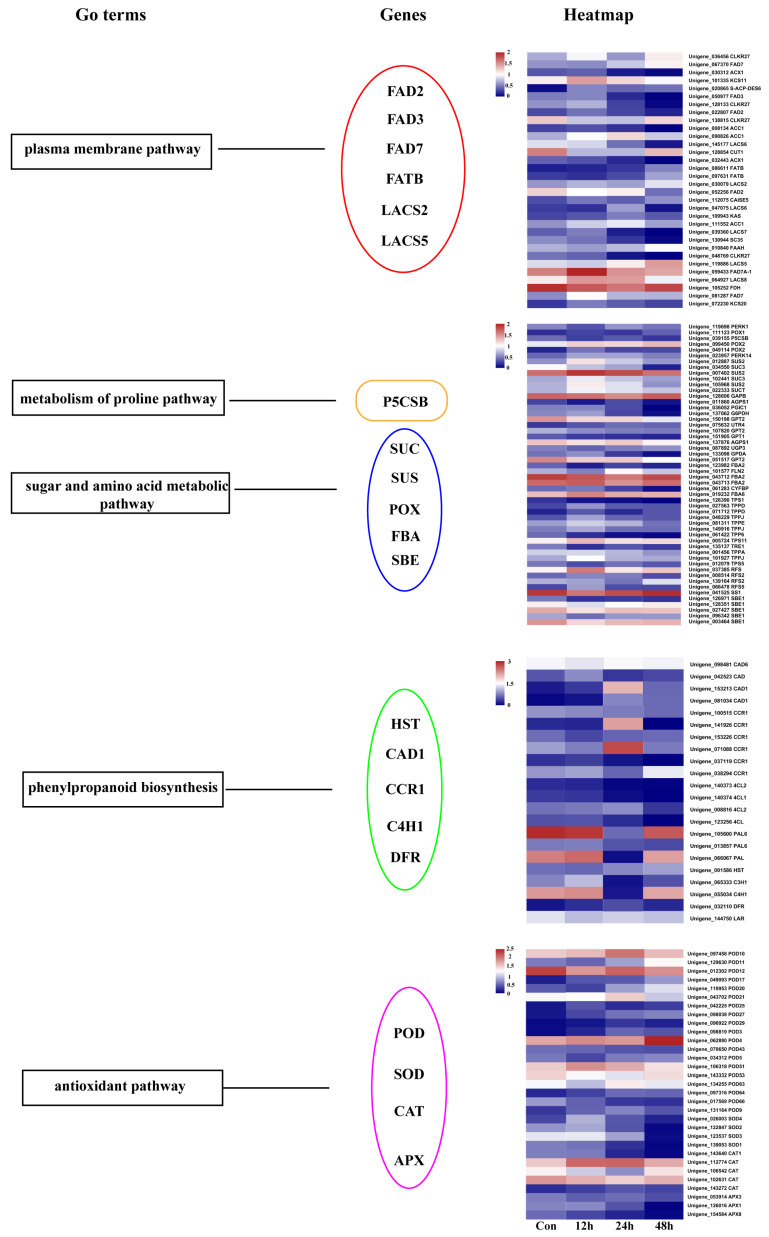
Heat map of DEGs implicated in the membrane system, metabolism of proline pathway, sugar metabolic pathway, phenylpropanoid biosynthesis, and antioxidant pathway.

The phenylpropanoid biosynthesis pathway is an important upstream pathway for the synthesis of phenolics, flavonoids, lignin, and other stimulatory metabolites in plants, and the increase in adverse conditions will protect cells from damage. Based on the KEGG enrichment results, the phenylpropanoid biosynthesis pathway is the metabolic pathway that is significantly enriched in DEGs in *L. caerulea* under salt stress; its related synthetic genes (HST, CAD1, and CCR1) were up-regulated (Figure 4), and the investigation of this regulatory pathway is critical for understanding the molecular mechanism of the response of *L. caerulea* to salt stress. In addition, the determination of flavonoids content in *L. caerulea* under salt stress revealed that with the extension of salt stress time, the flavonoid content also increased (Figure 1N).

Another KEGG enrichment of DEGs was starch and sucrose metabolism. In this research, six genes were present in sucrose metabolism with lower expression levels under salt stress (Figure 4); 41 DEGs were affected in the metabolism of glucose, fructose, galactose, kelpose, and cotton seed sugar. Seven genes were related to starch metabolism. Six genes participated in the metabolism of proline pathway, of which one proline synthase gene (P5CSB) was down-regulated under salt stress (Figure 4).

In this study, 31 antioxidant-related enzymes were involved in ROS, including 19 POD genes, 5 CAT genes, 4 SOD genes, and 3 APX genes (Figure 4). The expression of 12 POD genes was UR and 7 POD genes were DR after 12 h, 24 h, and 48 h of salt stress, and the expression of SOD-related genes did not change significantly after the stress. Four CAT genes were UR, and one POD gene was DR after 12 h, 24 h, and 48 h of salt stress, while the expression of APX gene showed a decreasing trend after salt stress for 12 h.

### 3.4. Different Salt Stress Durations Can Induce Different Metabolites in L. caerulea

Plant transcriptomics reveals the mechanisms underlying plant responses to environmental changes by analyzing variations in gene expression, while metabolomics provides a comprehensive view of the metabolites within plants, reflecting the ultimate outcomes of these gene expression changes. The integration of these two approaches allows for an in-depth investigation of plant growth and development, as well as responses to stress, from both the gene expression and metabolite change perspectives. It also helps elucidate the interactions and regulatory networks between gene expression and metabolite accumulation.

**Figure 5:**
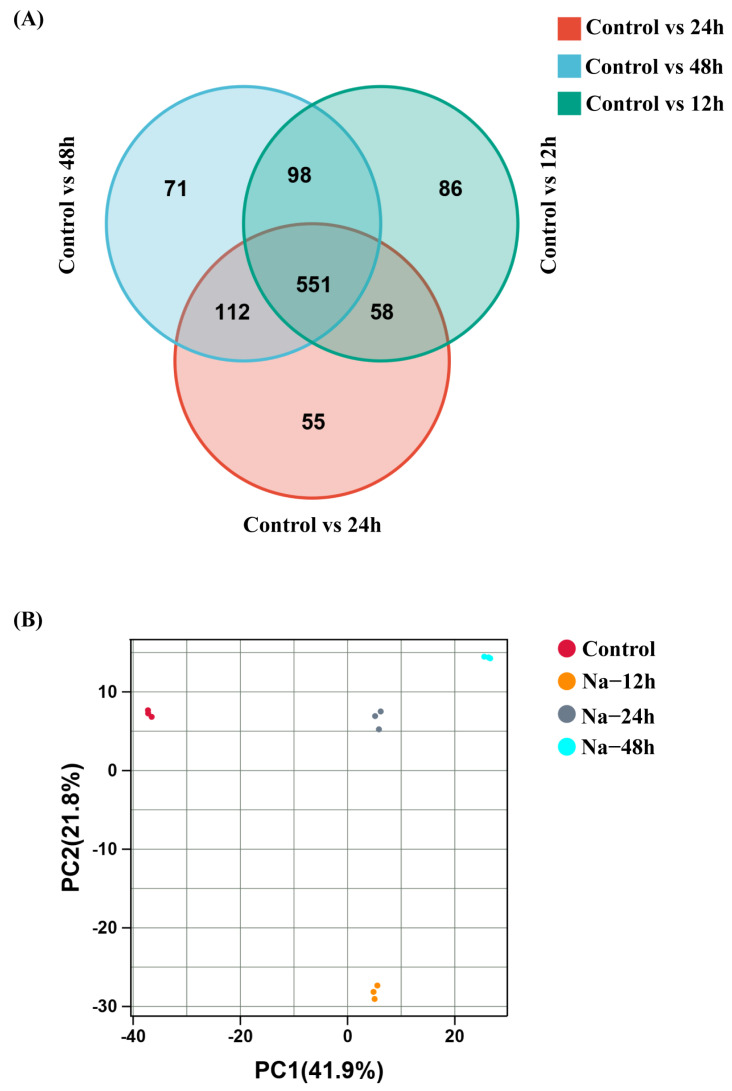
Venn diagram and PCA analysis of differential metabolites in *L. caerulea* under salt stress. (**A**) Venn diagram of differential metabolites at different time points under salt stress. (**B**) PCA analysis of all samples exposed to salt stress at four different (treatment) time intervals.

**Figure 6:**
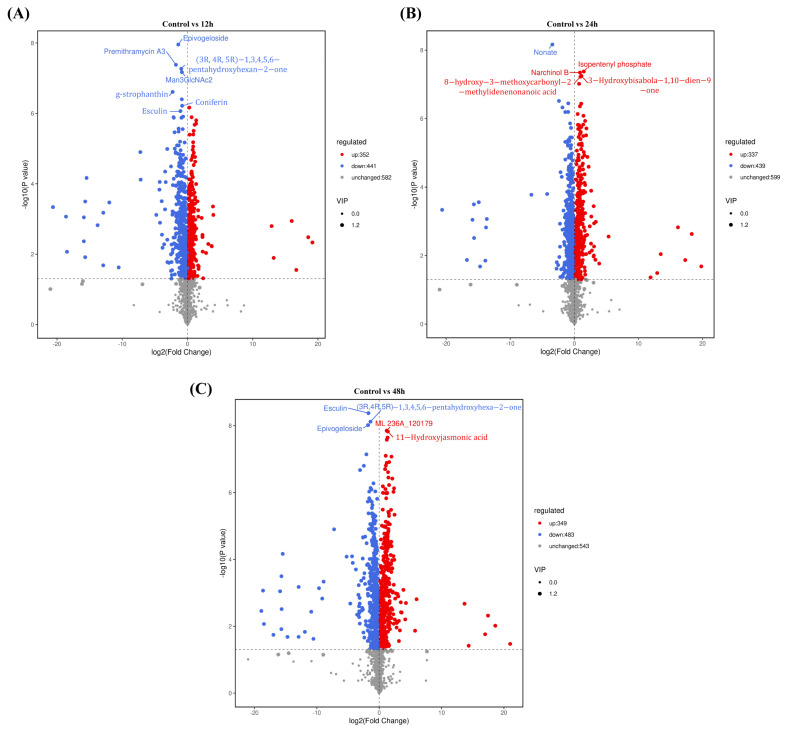
Volcano plots and VIP plots of differential metabolites in samples at different time points under salt stress treatment. The X-axis represents the fold change of metabolites, and the Y-axis represents the −log10 transformed *p*-value. A VIP value greater than 1.2 indicates significant differences in metabolites. The red dots and blue dots represent the up-regulation and down-regulation of metabolites, respectively. (**A**) The volcano and VIP plots show the differentially expressed metabolites between the control and 12 h salt stress. (**B**) The volcano and VIP plots show the differentially expressed metabolites between the control and 24 h salt stress. (**C**) The volcano and VIP plots show the differentially expressed metabolites between the control and 48 h salt stress.

To determine the effects of salt stress treatment (12 h, 24 h, and 48 h) on *L. caerulea* seedlings, we analyzed the seedlings after salt stress using liquid chromatography–mass spectrometry (LC-MS). A Venn diagram showed that 1375 metabolites were detected from the four differently treated *L. caerulea* samples, with the number of differential metabolites changing under different salt stress durations (Figure 5A). Principal component analysis (PCA) revealed that the differences in metabolites within each sample group were not significant, while the differences between groups were substantial (Figure 5B). The results of orthogonal partial least squares discriminant analysis (OPLS-DA) indicated that the samples from different treatments were clearly separated, with distinct clustering within groups, suggesting significant differences in metabolites between treatment groups. Volcano plots and VIP scores showed that after 12 h of salt stress, the relative concentrations of coniferin and esculin, which are phenylpropanoid metabolites, significantly decreased in *L. caerulea* seedlings, while the relative concentrations of lipid metabolites increased (Figure 6). After 24 h of salt stress, the relative concentrations of lipid metabolites in the seedlings continued to rise (Figure 6). After 48 h of salt stress, the relative concentration of esculin decreased further, and the relative concentration of lipid metabolites increased again (Figure 6). The above results indicate that after salt stress, plants undergo a series of physiological and biochemical changes within their tissues to counteract the effects of salt stress.

Using the KEGG database, we conducted a metabolic pathway enrichment analysis of the DAMs (Figure S2). Compared with the control, after 12 h of salt stress, 251 DAMs were annotated with KEGG, mainly participating in metabolic pathways such as Aminoacyl-tRNA biosynthesis, Cyanoamino acid metabolism, and Flavone and flavonol biosynthesis. After 24 h of salt stress, 226 DAMs were annotated with KEGG, primarily involved in pathways like Aminoacyl-tRNA biosynthesis, Purine metabolism, and Limonene and pinene degradation. After 48 h of salt stress, 246 DAMs were annotated with KEGG, mainly participating in pathways such as arginine and proline metabolism, Glycine, serine, and threonine metabolism, and arginine biosynthesis.

### 3.5. Correlation Between Transcriptomics and Metabolomics

To better understand the metabolic differences in *L. caerulea* at different time points after salt stress treatment, we focused on the significantly different metabolites mainly involved in the phenylpropanoid and fatty acid pathways, and we visualized the changes in metabolite accumulation using heatmaps. Additionally, to determine the relationship between metabolite changes and gene transcriptional regulation, we also measured the expression levels of these genes.

**Figure 7:**
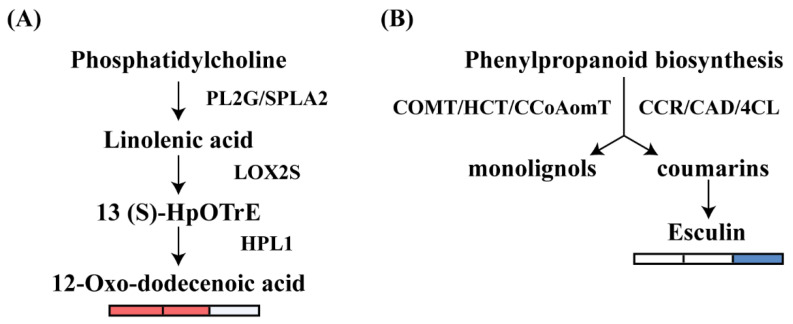
Schematic diagram of phenylpropanoid biosynthesis and fatty acid metabolism pathways in response to salt stress. The heatmap shows the analysis of differences in metabolite concentrations. In the heatmap, red indicates up-regulated metabolites, blue indicates down-regulated metabolites, and white indicates metabolites with no significant differences. (**A**) The fatty acid metabolism pathway begins with phosphatidylcholine, which is converted into Linolenic acid through the action of PL2G/SPLA2 enzymes. Linolenic acid is then further processed via LOX2S and 13 (S)-HpOTrE to produce 12-Oxo-dodecenoic acid. This compound is a key intermediate in the biosynthesis of various secondary metabolites. (**B**) Phenylpropanoids are synthesized from phenylpropanoid biosynthesis through the action of COMT/HCT/CCOAmT enzymes, leading to the formation of monolignols. These monolignols are then converted into coumarins via CCR/CAD/4CL enzymes, which are essential for the production of esculin, a significant secondary metabolite in this pathway. The first column of the matrix represents the differences in metabolite concentrations between control and 12 h. The second column of the matrix represents the differences in metabolite concentrations between control and 24 h. The third column of the matrix represents the differences in metabolite concentrations between control and 48 h.

After salt stress, *L. caerulea* produces many differential metabolites and DEGs. We conducted an integrated analysis of the transcriptome and metabolome to investigate the expression levels of differential metabolites and related genes in the phenylpropanoid and fatty acid pathways (Figure 7). In the fatty acid synthesis pathway, the relative content of 12-Oxo-dodecenoic acid significantly changed under salt stress at 12 h and 24 h, showing an upward trend. (Figure 7). To reduce salt stress-induced damage, the plasma membrane can only increase lipid mobility by controlling the level of unsaturated fatty acids. Under salt stress, 32 DEGs connected with fatty acid metabolism were present (Figure 4). Three of these omega-3 fatty acid desaturases, FAD2 (Unigene_052256, Unigene_022807), and FAD3 (Unigene_050977) were significantly down-regulated. Including FAD7 (Unigene_067370, Unigene_081287), FATB (Unigene_086611, Unigene_097631), LACS2 (Unigene_030079), LACS5 (Unigene_119886) were significantly up-regulated (Figure 4). By measuring MDA levels and relative electrolyte leakage rates, we found that the lower the MDA level, the better the cell membrane state. MDA levels and relative electrolyte leakage were significantly increased at 12 h, 24 h, and 48 h compared to the control of the salt stress treatment.

In the phenylpropanoid metabolic pathway, the contents of coniferin and esculin both decreased during the period of salt stress from 12 h to 48 h. This observation is consistent with the KEGG enrichment results (Figure 4). Additionally, the study revealed that the flavonoid content in *L. caerulea* increased with the extension of salt stress time, which may reflect an adaptive response of the plant under prolonged stress.

## 4. Discussion

When plants respond to salt stress, the expression of salt stress-related genes in plants will change, and a series of complex regulatory mechanisms will be formed within the plant to counteract the effects of salt stress [62]. Changes in gene expression levels can significantly affect the synthesis of transcriptional regulatory proteins within the organism. Alterations in protein synthesis directly impact the production of metabolites and other regulatory mechanisms [63]. In this study, based on the results of the physiological changes of *L. caerulea* under salt stress, the transcriptome and metabolome of *L. caerulea* were sequenced under different salt stress treatment times, and the expression of genes related to salt stress in *L. caerulea* was analyzed through the transcriptome data, as well as the gene regulatory pathways and networks, in order to explore the potential of salt tolerance of *L. caerulea* and provide technical support for the cultivation of salt-tolerant varieties and the efficient use of saline and alkaline land.

This study examined the physiological responses of blue honeysuckle to salt stress, analyzing variations in the transcriptome and metabolome across different durations of exposure. The study identified differentially expressed genes (DEGs) and metabolic pathways that regulate blue honeysuckle’s response to salt stress.

Under salt stress, the rice mutant with the knockout of the *OsPP2C68* gene exhibits enhanced salt tolerance and reduced stomatal conductance, with a significant up-regulation of genes related to stomatal closure (such as *OsDREB6*, *OsHKT1*, and *OsNAC9*) [64]. Our results showed that, under salt stress, genes related to stomatal closure were up-regulated, which is consistent with the findings of Wang et al. These physiological and transcriptional changes indicate that stomatal closure plays an important regulatory role in the plant’s response to salt stress.

During unfavorable conditions, plants generate excessive ROS [65,66,67]. ROS play dual functions in cells: they act as the signaling molecules when they are at low levels and will induce an oxidative stress (OS) response that damages macromolecular substances like DNA, RNA, proteins, and lipids when present in a high amount. Thus, regulating ROS at appropriate levels during abiotic stress conditions is important [68,69]. In *L. caerulea* (blue honeysuckle), the overexpression of genes such as *LcNAC73*, *LcMYB5*, *LcMYB71*, and *LcMYB90* enhances stress tolerance by increasing the activities of enzymes like POD, CAT, and APX [35,36,37]. Similar overexpression studies of analogous genes in other plants have also made significant progress. *PvNAC1* promotes biomass and salt tolerance in switchgrass (*Panicum virgatum* L.) by lowering Na^+^ accumulation and improving ROS scavenging [70]. Stomatal morphological changes, such as variations in the length/width ratio, are typically associated with stomatal opening and closing capabilities, gas exchange efficiency, and water use efficiency [71]. Under short-term stress conditions, stomatal morphological changes may serve as a rapid adaptation mechanism for plants to cope with environmental changes [72]. For instance, narrower or shorter stomata may help reduce water evaporation, thereby enhancing the plant’s heat resistance [38]. The overexpression of *NtGCN2* in tobacco enhances drought tolerance by modulating proline levels, the ROS scavenging ability, and stomatal opening and closing [39]. These morphological changes not only reflect the immediate response of plants to short-term stress but may also exert long-term effects on plant growth and survival. However, there are currently no applications of gene knockout in *L. caerulea*. In this investigation, we discovered that the activities of enzymes linked to ROS scavenging capacity and other enzymes were changed in *L. caerulea* at different (treatment) time intervals (Figure 1), indicating that salt stress affects the expression of genes such as POD, CAT, and APX. Combining the results of overexpression or gene knockout in other species, we found that homologous genes exhibit both conservation and specificity across different species [40]. This provides theoretical support for the future development of new varieties through gene editing.

To mitigate the changes in osmotic potential caused by salt stress, plants typically accumulate starch and soluble sugars to maintain osmotic balance (Figure 1). The results are similar to those of Zhang et al. [73]. Our study showed that soluble sugar and starch content of *L. caerulea* increased significantly under salt stress, suggesting that the sugar metabolism pathway may play an important role in plant response to salt stress.

Fatty acids are a group of unique bioactive substances that play an important role in regulating plant growth and development, as well as in resisting abiotic stresses [74,75]. Many genes related to fatty acid synthesis or degradation are associated with plant responses to abiotic stresses. [76,77,78]. In this study, we found that the MDA content and electrolyte leakage of *L. caerulea* increased under salt stress (Figure 1). The results are similar to those of Sun et al. [76]. These findings imply that the fatty acid metabolic pathway may be related to plant cell membrane damage under salt stress in *L. caerulea*, and two lipid-related differential metabolites were identified as having been altered.

The secondary metabolites of the phenylpropanoid metabolic pathway, such as phenylpropanoid biosynthesis, are related to the formation of lignin in plant cell walls and are the main substances for plants to resist adverse environmental stresses [79]. The phenylpropanoid pathway responds to salt stress by regulating the synthesis of free radicals, the integrity of the cell membrane, and the thickness of the cell wall. Yu found that overexpression of *SiMYB16* in rice could increase the content of flavonoids and lignin to cope with salt stress [80]. In *Arabidopsis* and *Marchantia polymorpha*, G proteins enhance plant salt tolerance by strengthening the phenylpropanoid pathway and the abscisic acid response [81]. In this study, a total of four up-regulated expression-related genes and two DAMs were found to be altered in the phenylpropanoid biosynthesis pathway, suggesting that the response of *L. caerulea* to salt stress may be related to the positive regulation of phenylpropanoid biosynthesis (Figure 7).

In this study, the transcriptome data of *L. caerulea* under salt stress conditions were analyzed through RNA-Seq, and 4081, 4042, and 4403 DEGs were obtained at 12 h, 24 h, and 48 h, respectively. The DEGs were significantly enriched in the antioxidant response, the MAPK response, phytohormone signaling, and the phenylpropanoid biosynthesis pathway. The changes in these DEGs are closely related to the alterations in plant physiological indicators. The high expression levels of SODs, PODs, and CATs contributed to the conference on salt tolerance in *S. portulacastrum*, indicating the same trend as the physiological level [82]. The DEGs related to MAPKs were enriched in *M. laxiflora* seedlings in response to Cd stress. The expression changes of these genes indirectly affect the activity of antioxidant enzymes and cell damage indicators by regulating plant hormone levels and MAPK signaling pathways [83]. Phenylpropanoid biosynthesis is also a source of antioxidants. The overexpression of *NtERF13a* in tobacco enhances tolerance to abiotic stress and the synthesis of phenylpropanoid compounds by upregulating the expression of *NtHCT*, *NtF3′H*, and *NtAN* [84]. In addition, the increase in phenylpropanoid content may enhance cell wall stability, thereby alleviating cell damage and reducing the content of malondialdehyde MDA and electrolyte leakage. Our results are consistent with previous studies. The enrichment of DEGs in the transcriptome in antioxidant responses, MAPK signaling, plant hormone signaling, and phenylpropanoid biosynthesis pathways is in line with the trends observed in plant physiological indicators, such as antioxidant enzyme activity, phenylpropanoid content, soluble sugar content, proline content, malondialdehyde content, and electrolyte leakage. The findings of this work will give more evidence for future research on the mechanism of salinity tolerance for *L. caerulea*, as well as provide the material base for the cultivation of salt-tolerant cultivars. The study in this paper faced some limitations: (1) The range of salt stress concentrations used in this study was limited and may not have fully covered the salt stress conditions that *L. caerulea* experiences in natural environments. Additionally, the experiments were conducted under greenhouse conditions, lacking consideration of complex field environmental factors such as drought and high temperature. (2) Genes with significant differences were not selected from the transcriptome for gene function identification. (3) Different *L. caerulea* varieties with varying salt tolerance abilities should be selected for comparative studies to analyze their physiological, transcriptomic, and metabolomic differences under salt stress, thereby revealing the molecular mechanisms underlying the differences in salt tolerance.

In the future, we aim to identify DEGs in *L. caerulea* and employ molecular techniques to investigate the regulatory network associated with resistance. This will help elucidate the salt tolerance mechanism of *L. caerulea* under salt stress conditions. Additionally, we plan to identify genes with stress-resistant properties, conduct transgenic breeding studies, and enhance germplasm to facilitate the expansion of *L. caerulea* cultivation areas and foster the growth of the *L. caerulea* industry.

## 5. Conclusions

**Figure 8:**
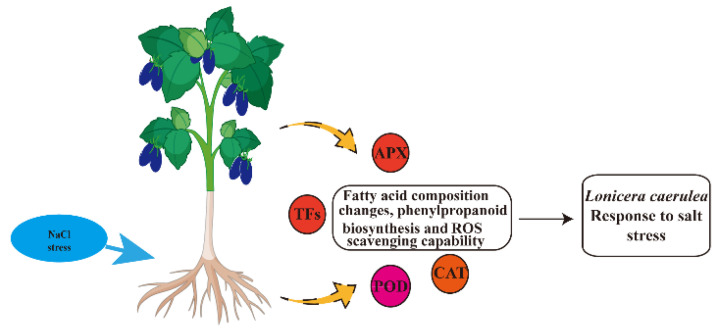
Establishment of a working model for *L. caerulea* in response to salt stress.

We proposed a model for salt tolerance in *L. caerulea* (Figure 8). Salt stress, as an important abiotic stressor, can activate fatty acid synthesis and phenylpropanoid metabolic pathways, leading to the activation or repression of relevant genes. *L. caerulea* was able to activate antioxidant enzyme systems, such as superoxide dismutase, catalase, and ascorbate peroxidase, to scavenge ROS and reduce the damage of oxidative stress under salt stress. *L. caerulea* adapts to and resists the adverse effects of salt stress by regulating physiological changes such as antioxidant enzyme activities and proline content under salt stress. The above genes lead to changes in stomata, fatty acid metabolism, and phenylpropanoid biosynthesis metabolism.

## Data Availability

The data presented in the study have been deposited into the NCBI repository under accession number PRJNA1023716. All data supporting the findings of this study are available within the paper and within its Supplementary Files. The accession numbers of sequence data from this article can be found in the GenBank data libraries (https://www.ncbi.nlm.nih.gov/search) (accessed on 13 December 2023): Unigene_002872 (OR887655): https://www.ncbi.nlm.nih.gov/nuccore/OR887655 (accessed on 13 December 2023), Unigene_006964 (OR887656): https://www.ncbi.nlm.nih.gov/nuccore/OR887656 (accessed on 13 December 2023), Unigene_009130 (OR887657): https://www.ncbi.nlm.nih.gov/nuccore/OR887657 (accessed on 13 December 2023), Unigene_012269 (OR887658): https://www.ncbi.nlm.nih.gov/nuccore/OR887658 (accessed on 13 December 2023), Unigene_015249 (OR887659): https://www.ncbi.nlm.nih.gov/nuccore/OR887659 (accessed on 13 December 2023), Unigene_020376 (OR887660): https://www.ncbi.nlm.nih.gov/nuccore/OR887660 (accessed on 13 December 2023), and Unigene_020969 (OR887661): https://www.ncbi.nlm.nih.gov/nuccore/OR887661 (accessed on 13 December 2023).

## Supplementary Materials

The following supporting information can be downloaded at https://www.mdpi.com/article/10.3390/biology14060641/s1, Supplemental Figure S1. Demonstrating the reliability of RNA-seq data; Supplemental Figure S2. KEGG enrichment map of DAMs. (A–C) The bigger the enrichment factor, the greater the importance of DAMs in the pathway. The color of the circle symbolizes the q-value. The larger the q-value, the less credible the importance of the containing of DAMs in the pathway. A represents the control vs. 12 h, B represents the control vs. 24 h, and C represents control vs. 48 h; Supplemental Table S1. The primer sequences used in the PCR.
