# Supplementary figures and images for: Transcriptome and Metabolome Analyses of the Salt Stress Response Mechanism in Lonicera caerulea
Source: Biology (Basel). 2025 May 31;14(6):641. doi: 10.3390/biology14060641 (PMC12189683; doi:10.3390/biology14060641)

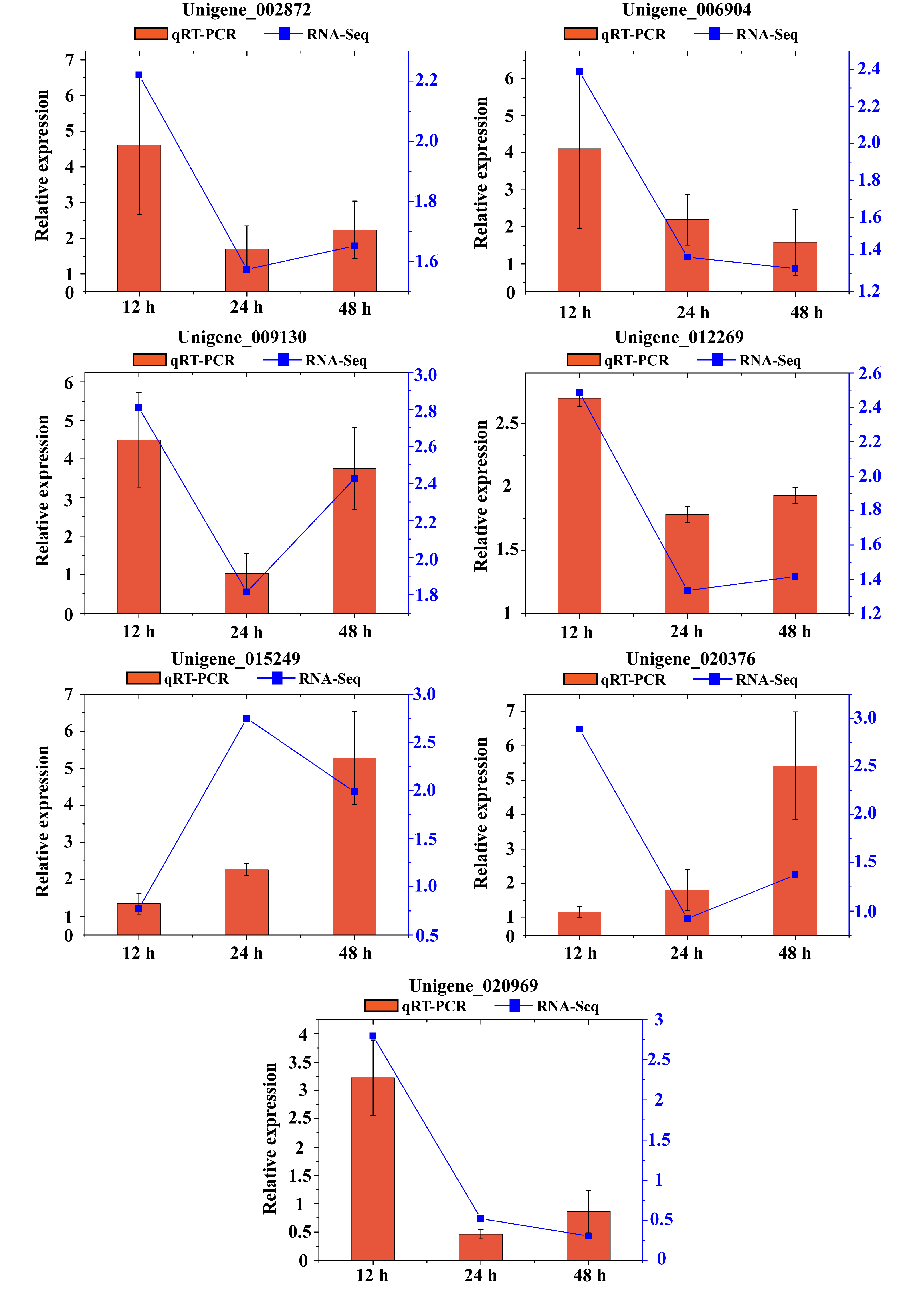

Supplement: Supplementary file 1 [file biology-14-00641-s001.zip › Figure S1.tif]

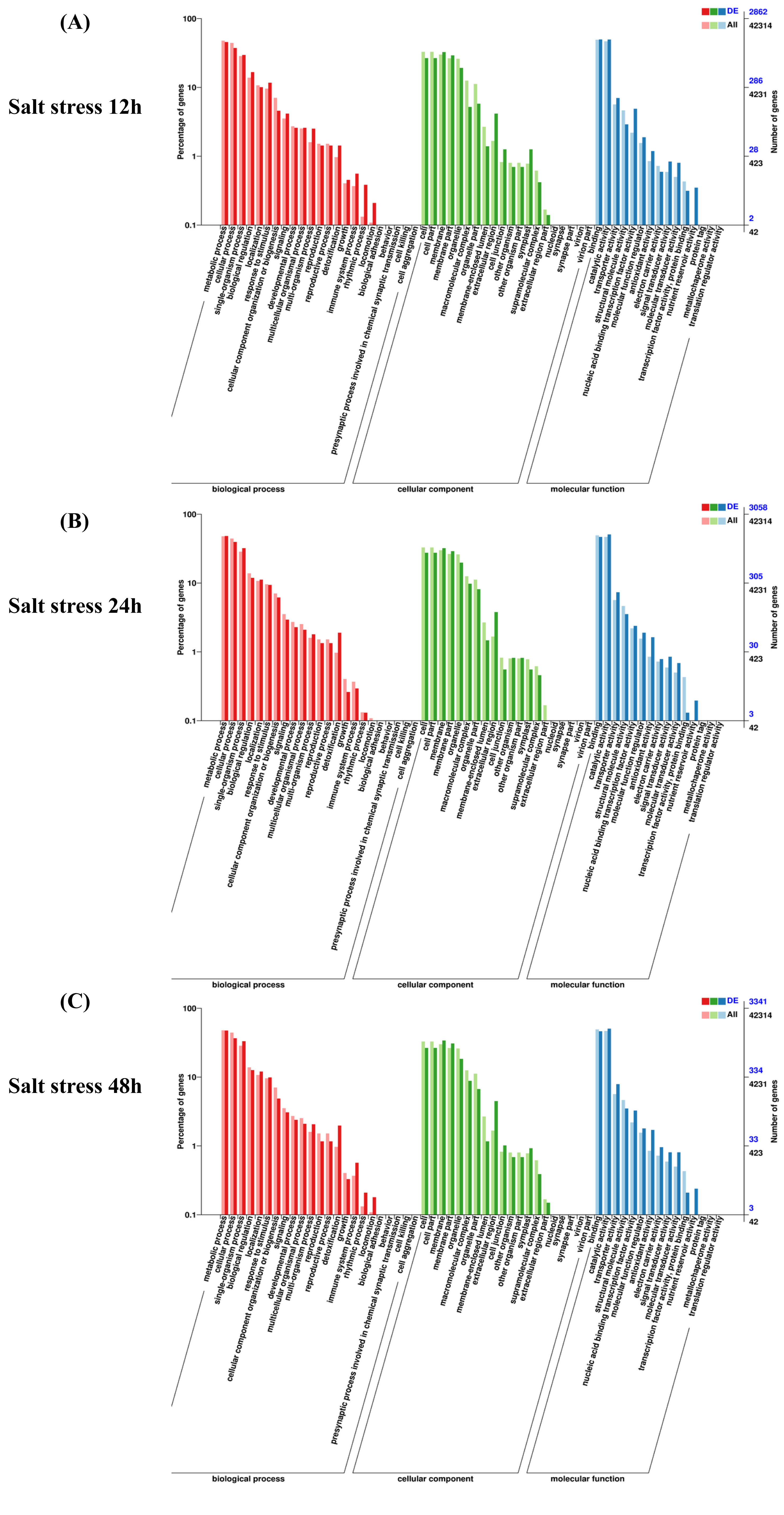

Supplement: Supplementary file 1 [file biology-14-00641-s001.zip › Figure S2.tif]
